# The xenotropic murine leukemia virus-related retrovirus debate continues at first international workshop

**DOI:** 10.1186/1742-4690-7-113

**Published:** 2010-12-22

**Authors:** Jonathan P Stoye, Robert H Silverman, Charles A Boucher, Stuart FJ Le Grice

**Affiliations:** 1 MRC National Institute for Medical Research, The Ridgeway, Mill Hill, London NW71AA, UK; 2 Department of Cancer Biology, Lerner Research Institute, Cleveland Clinic, 9500 Euclid Avenue, Cleveland, OH 44195, USA; 3 Department of Virology, Erasmus MC, University Medical Centre Rotterdam, Rotterdam, The Netherlands; 4 HIV Drug Resistance Program, National Cancer Institute, Frederick, MD 21702, USA

## Abstract

The 1^st ^International Workshop on Xenotropic Murine Leukemia Virus-Related Retrovirus (XMRV), co-sponsored by the National Institutes of Health, The Department of Health and Human Services and Abbott Diagnostics, was convened on September 7/8, 2010 on the NIH campus, Bethesda, MD. Attracting an international audience of over 200 participants, the 2-day event combined a series of plenary talks with updates on different aspects of XMRV research, addressing basic gammaretrovirus biology, host response, association of XMRV with chronic fatigue syndrome and prostate cancer, assay development and epidemiology. The current status of XMRV research, concerns among the scientific community and suggestions for future actions are summarized in this meeting report.

## Introduction

In 2006, Urisman *et al*. [1] described the identification and characterization of a novel gammaretrovirus, xenotropic murine leukemia virus-related virus (XMRV), in a small number of prostate cancers. Subsequent studies of Schlaberg *et al*. [2] suggested that XMRV might have a broader distribution, and was present in both prostate cancer patients and benign controls. XMRV is very closely related to endogenous proviruses found in inbred (laboratory) mice, some of which cause lymphoma and other diseases in mice. Due to the lack of functional receptor *Xpr1*, this virus does not replicate in most inbred mice, but grows well in human prostate cancer cell lines. Interest in XMRV has recently intensified following the work of Lombardi *et al*. [3], who detected XMRV in chronic fatigue syndrome (CFS) patients in clusters of cases in Nevada and Florida-South Carolina. Virus could be detected through both antibodies in serum and proviral DNA in peripheral blood mononuclear cells (PBMCs), and could easily be cultured from PBMCs and plasma. However, although these and related studies demonstrated an association of XMRV infection with at least two human diseases, causality was not established.

Despite the significant increase in XMRV-related publications over the last 24 months, the research community has failed to reach consensus on the origin of this virus, its causative (or passenger) role in disease pathology, and the extent to which it is prevalent in the human population. On the contrary, the numbers of studies identifying XMRV in humans [1-6] are presently outweighed by reports from laboratories throughout the world that have failed to detect the virus [7-15] which have now added to an increasing sense of confusion. Central to this has been the lack of standardized nucleic acid-based or serological methods for detecting viral nucleic acid and antibodies, respectively, as well as "gold standard" reference samples with which individual groups can judge the selectivity and sensitivity of their protocols. The 1^st ^International Workshop on XMRV was therefore convened at the National Institutes of Health, Bethesda, MD on September 7/8, 2010, with a goal of providing a public forum to discuss these and related issues, including increasing concerns regarding mouse DNA contamination, methods of sample handling and storage, use of antiretrovirals currently available for HIV therapy, and progress in developing standard PCR and serological reagents. In his introductory remarks, NIH Director Dr. Francis Collins urged the 225 attendees to maintain a healthy skepticism on potential causative roles of XMRV, indicating that a solution to this conundrum requires an interdisciplinary and synergistic effort from researchers in both the prostate cancer and CFS arenas. This report summarizes overviews and research findings presented during the 2-day International Workshop.

### Gammaretrovirus Biology

**J. Coffin **(Tufts University School of Medicine, Boston, Massachusetts) opened the Workshop by providing background information on XMRV and the endogenous viruses of mice, summarizing the basic properties of endogenous retroviruses and the original studies with XMRV before proceeding to examine in more detail proviruses in the genomes of mice and their effects on their hosts. Experiments in his laboratory have characterized xenotropic, polytropic and modified polytropic endogenous proviruses, their distribution across the mouse genome, co-evolution with different species of mice, and relationship to viruses associated with prostate cancer and chronic fatigue syndrome. Dr. Coffin's concluding statements set the tone for subsequent discussions of the Workshop. Uppermost in his concerns were (i), conflicting reports on association with diseases (ii), lack of insight into potential pathogenic mechanisms (iii), assay sensitivity used for detecting XMRV and related viruses (iv), the well documented infection of human cells passaged through nude mice by xenotropic MLV possibly initiating further spread, and (v) ubiquitous presence of mice and mouse products likely containing multiple MLV sequences. The magnitude of the problem was illustrated by considering a swimming pool into which a drop of mouse blood was introduced, after which every milliliter of water would contain enough DNA to give a positive signal using the current ultrasensitive PCR techniques.

**S. Chow **(University of California, Los Angeles, California) described studies of the incidence of XMRV and related MLV in healthy donors and patients with prostate cancer in two cohorts, one in the U.S. (UCLA) and the other in China (Second Affiliated Hospital, Hangzhou, Zhejiang and Ningbo Blood Center, Ningbo, Zhejiang) using an RT-PCR approach with three different primer sets. Individuals were considered positive if one out of the three tests yielded consistent positives. Perhaps surprisingly, an equal frequency of positives was seen in the patient and control groups, and there was a higher incidence of XMRV or MLV-related virus sequences associated with increasing age. An association with the RNase L R462Q mutation previously linked with prostate cancer was not confirmed. *Env *primers yielded the most positive results; including examples of XMRV, xenotropic, polytropic and modified polytropic sequences. No examples of the 24-nt deletion in the *gag *leader region characteristic of XMRV were detected with fragments amplified using *gag *primers. Dr Chow subsequently described experiments to identify XMRV-host cell junctions in samples from CFS patients. Such junction fragments were only found in two XMRV-positive, patient-derived cell lines [16,17]. The sample size of XMRV integration sites in tumors is currently insufficient to detect common integration sites with which to assess the role of insertional mutagenesis as an oncogenic mechanism during XMRV infection.

**A. Wlodawer **(National Cancer Institute (NCI), Frederick, Maryland) opened his presentation by pointing out the contribution of drugs targeting HIV-1 protease to highly-active antiretroviral therapy, and the crucial role of structure-based drug design in their development. Dr. Wlodawer reported the 2Å crystal structure of XMRV protease, which is responsible for processing protein polyprotein precursors during virus maturation. As with related retroviral proteases, the XMRV protein forms a homodimer, but despite overall similarities, the XMRV and HIV-1 proteins are quite different, particularly at the dimer interface. Overall, the structure resembles an internal domain from a human ubiquitin receptor protein (Ddi1) that may function proteolytically during regulated protein turnover in the cell. Recombinant XMRV protease showed a tendency for self-digestion, an observation that will presumably be used in the development of XMRV protease inhibitors. An account of this study will appear in *Nature Structural and Molecular Biology*.

**O. Cingoz **(Tufts University, Boston, Massachusetts) described different approaches to identify a possible source of XMRV in mice. Sequence comparisons were conducted to design a pair of PCR primers, spanning (i), a unique 2-nt insertion in the viral LTR and (ii) the 24-nt deletion of the *gag *leader, allowing detection of XMRV against a background of closely related MLVs. Screening over 70 inbred laboratory and wild derived mice failed to identify an endogenous provirus with the predicted fragment. However *in silico *analyses showed that one or more proviruses carrying the 24-nt deletion is present in several mouse strains, an observation that was confirmed by single genome PCR amplification and Southern blotting experiments using an oligonucleotide probe spanning the deletion. This probe reacts with a single provirus in these strains whose similarity with XMRV remains to be determined. Together these observations strengthen the argument for a murine origin of XMRV with recombination or mutation providing the LTR specific change. Dr. Cingoz concluded by describing a highly sensitive assay for detecting mouse DNA contamination using primers directed against Intracisternal A-type particle (IAP) sequences, which are present at an estimated 1000 copies per haploid genome [18]. An assay with such sensitivity, possibly complementing one directed against mouse mitochondrial DNA, would guard against contaminating DNA in future PCR studies designed to detect XMRV and related viruses in human samples.

The concluding presentation of **R. Molinaro **(Emory University, Atlanta, Georgia) described a novel gene product encoded by XMRV, translated from a doubly spliced *env *mRNA of 1.2 kb and comprising an 11kD portion of the C-terminal region of the Envelope polyprotein. Expression studies with a GFP fusion protein revealed a punctate pattern of fluorescence present in both nucleus and cytoplasm. These studies are consistent with, but do not prove, a possible role for the novel protein in the export of unspliced viral RNA from the nucleus of XMRV-infected cells.

### Host Response

The Host Response session was opened by **R. Silverman **(Cleveland Clinic, Cleveland, Ohio), who discussed the linkage of hereditary prostate cancer with mutations in the ribonuclease L (RNase L) gene and discovery of XMRV [1]. In 86 patients, the finding that 8/20 were homozygous for the QQ mutation in RNase L suggested a strong correlation [1], confirmed in one study [4] but not in others [2,5]. Data were presented showing that RNase L inhibited XMRV replication in cell culture. Electron microscopy identified an enveloped retrovirus similar to MLV. A rhesus macaque study with Francois Villinger and collaborators at Abbott Diagnostics (to be described), showed that XMRV trafficked to prostate epithelium within 6-7 days post-infection, but was observed only in stromal cells after 291 days. Similarly, in human prostate cancer tissues XMRV was observed only in a small number of stromal cells [1]. The XMRV DNA in macaque PBMC in vivo was mutated by APOBEC3, relating to the subsequent talk by K. Bishop. The androgen receptor element in the XMRV LTR U3 region was shown to be sensitive to dihydroxytestosterone (DHT) *in vitro*, and DHT stimulated virus replication *in vitro *[19]. Dr. Silverman concluded with a statement that a causal link of XMRV to any human disease remains to be established.

In view of the increasing interest in cellular inhibitors of retroviral replication, **K. Bishop **(National Institute for Medical Research, UK) provided an overview of restriction factors and their impact on XMRV replication [20]. Human APOBEC3G, a cytidine deaminase, which potently inhibits HIV replication through lethal G -> A hypermutation, and to a lesser extent the related APOBEC3F, were also shown to inhibit XMRV replication. However, while the HIV accessory protein Vif counters APOBEC-mediated deamination by targeting it for proteasomal degradation, XMRV lacks the counterpart. How XMRV achieves such "resistance" is presently unclear. Tetherin (CD317/Bst2), a Type II membrane protein that localizes to multiple membrane compartments, crosslinks nascent virions to the plasma membrane, preventing release of a variety of retroviruses, filoviruses, gammaherpesviruses and arenaviruses. XMRV was likewise sensitive to human, simian and murine tetherins. While HIV-1 Vpu counters tetherin-mediate XMRV restriction in HeLa cells, the absence of such an accessory protein in XMRV again begs the question of how host restriction is bypassed. Also, XMRV env cannot counteract tetherin. Finally, XMRV is not sensitive to restriction by the intrinsic immune factor TRIM5alpha, which can mediate an early block to HIV-1 replication. However, XMRV is restricted by the mouse specific restriction factors, Fv1^n ^and Fv1^b^. Understanding how XMRV evades host restriction factors in the course of natural infection is clearly an important issue if developing antiviral strategies becomes a priority.

Although the XMRV field is in its infancy, **E. Sparger **(University of California at Davis, California) highlighted issues that must be better understood when considering vaccine development. These included the mode(s) of transmission, pathogenesis in the host and immune correlates for control of virus replication. Dr. Sparger's comments were based on the success of vaccinating domestic cats against feline leukemia virus (FeLV), a related gammaretrovirus identified in 1964. Common features of FeLV and XMRV include their potential association with immune suppression, disease of the central nervous system, and induction of cancer. Successful strategies included whole inactivated virus, recombinant surface glycoprotein, subunit vaccines and nonadjuvanted canarypox-vectored live vaccine (ALVAC), with efficacy rates of 44% - 100% reported. Reiterating the cautious note that pathogenesis and immune correlates for XMRV must be thoroughly characterized in order to inform vaccine design, Dr. Sparger concluded by suggesting that newer and more novel approaches (e.g. vector systems, molecular adjuvants, inclusion of multiple modalities) should further increase the likelihood of success.

With the goal of establishing an animal model to study XMRV dissemination, tissue tropism and pathogenicity, **F. Villinger **(Emory University, Atlanta, Georgia) summarized a collaborative study in which XMRV-infected rhesus macaques were followed for various periods of time post-infection and euthanized during acute infection or during chronic infection 146 and 289 days post-inoculation [21]. Animals were monitored for immune parameters and viral replication as well as extensive tissue collections and *in situ *analyses performed at necropsy. Animals showed transient viremia and induction of antibodies as well as infection of prostate, spleen, liver, lymph nodes, lung and jejunum. No evidence of pathogenesis was observed during the 9-month follow-up, together with antibody responses that rapidly declined after infection and mostly undetectable cell mediated immune responses, suggesting limited antigenic stimulation. However, detailed *in situ *analysis of various organs and tissues detected virus replication at various times post-infection. Demonstration of XMRV replication in reproductive organs (prostate, seminal gland, testis as well as vagina and cervix) suggested a potential for sexual transmission. In cautioning that expansion of the model is urgently needed, this study provided a valuable model of human XMRV infection to assess long-term chronic infection, pathogenesis, immunity and for validating potential vaccines.

The use of Gairdner's Shrewmouse, *Mus pahari*, as a small animal model of XMRV infection was presented by **Y. Ikeda **(Mayo Clinic, Rochester, Minnesota). The susceptibility of *Mus pahari *cells to XMRV is due to their novel receptor as previously described by C. Kozak and co-workers [22]. The Kozak laboratory also showed that no wild mouse is resistant to xenotropic virus and several laboratory mouse strains are susceptible to X-MLVs [23,24]. The Ikeda laboratory showed that *M. pahari *fibroblasts support XMRV replication *in vitro*, while inoculated mice demonstrated high levels of neutralizing antibodies 2 weeks post-infection. XMRV proviral DNA was found mainly in blood, spleen and brain, suggesting the virus was lympho- and neuro-tropic in *Mus pahari *[25]. Despite some practical difficulties (including small litters, relatively small spleen and a lack of inbred strains), the *Mus pahari *model showed promise.

To uncover additional determinants of virus entry and identify entry restrictions that could modulate trans-species transmissions, **C. Kozak **(NIAID, Bethesda, Maryland) examined evolution of *Xpr1 *in rodent species and the co-evolution of *Xpr1 *and xenotropic/polytropic MLVs (X/P-MLVs) in *Mus *species, extending this analysis to non-rodent species. Ten distinct phenotypes were identified, distinguished by resistance to different X/P-MLVs, of which four were known *Xpr1 *variants in *Mus *and a novel fifth allele was identified in *Mus molossinus *and *Mus musculus*. The geographic and species distribution of the five functional *Xpr1 *variants in *Mus *and their evolutionary association with endogenous X/P-MLVs were described. Specific residues important for mouse X/P-MLV entry were demonstrated by mutational analysis, which also indicated that, while XMRV relies on X-MLV entry determinants, it uniquely requires at least one additional residue. In demonstrating the highly polymorphic nature of the *Xpr1 *receptor, Dr. Kozak emphasized that, although all mammals carry functional receptors, these differ in their ability to allow entry of the various human or mouse derived viruses, reflecting sequence substitutions or deletions in the two extracellular loops that carry receptor determinants.

### XMRV and Prostate Cancer

In his introductory presentation, **E. Klein **(Cleveland Clinic, Cleveland, Ohio) addressed four questions: 1) why is prostate cancer important? 2) is prostate cancer an infectious disease? 3) what is the role of XMRV in prostate cancer? 4) what are the implications? Risk factors for prostate cancer include age, race, family history and genetic factors that remain largely undefined. Infections account for several types of cancers, but it is unknown if infectious agents contribute to prostate cancer. However, mutations in genes involved in the host response to infections or in immunity (e.g., *RNASEL*, *MSR1 *and *TLR4*) are associated with prostate cancer in humans. The *RNASEL *(*HPC1*) association is seen in multiple affected family members [26]. RNase L R462Q has reduced enzyme activity and doubles the risk of prostate cancer when homozygous [27]. XMRV was discovered in such men (QQ genotype) with prostate cancer [1]. Published confirmatory studies of XMRV in prostate cancer were described [2,4,28], although only one suggested correlation with the *RNASEL *QQ genotype [4]. Possible reasons for studies failing to detect such an association [12] are that XMRV may not be truly associated with human disease, technique differences (e.g. PCR details and unrecognized sequence variations), and geographical distribution of the virus. Pathways to viral oncogenesis include insertional mutagenesis, proinflammatory effects, oncogenic viral proteins, immune suppression and altered epithelial/stromal interactions. For instance, cancer associated fibroblasts (but not normal fibroblast) cause initiated epithelial cells to form large tumors in mice. The implications of XMRV in prostate cancer include a potential biomarker for aggressive tumors [2]. In this regard, XMRV RNA was detected in a subset of expressed prostate secretion (EPS) specimens from prostate cancer patients. Dr. Klein closed by suggesting that if XMRV is shown to be a cause of prostate cancer it could lead to a vaccine, such as the HPV vaccine used to prevent cervical cancer.

**I. Singh **(University of Utah, Salt Lake City, Utah) reviewed her work on the role of XMRV in prostate cancer [2] and of antiretroviral drugs on XMRV infections in cell culture [29]. Compelling reasons for studying XMRV included a large number of prostate cancer deaths, and a causal role for XMRV could spur new methods for prevention, biomarkers for disease, help in resolving difficult cases and antiretroviral therapy. Rabbit antisera to XMRV propagated in human 293T cells was used in immunohistochemistry (IHC) experiments to probe human prostate tumor tissue sections (23% of which were positive). Infected cells were almost all of the malignant epithelial type, including clusters of such cells. In contrast, qPCR showed 6% were XMRV positive. Differences between the two methods were attributed to very low viral loads, sampling differences, and varying proportions of XMRV-infected cells. XMRV was associated with higher grades of prostate cancer, but not tumor stage or age at diagnosis. Since association between XMRV detection and the *RNASEL *SNP for R462Q could not be verified, the entire population may be susceptible to XMRV infection. Lessons learned include that very small amounts of virus are present, contamination from mouse tissues can occur, different sections of the same tumor may have different amounts of virus, and that XMRV detection by IHC does not work well in tissue microarrays.

**J. Petros **(Emory University, Atlanta, Georgia) described XMRV variations in prostate cancer cases, pointing out that there are relatively few SNP variations between reported XMRV sequences and only limited full-length XMRV genome sequences in the public domain. Evidence of XMRV in prostate cancer cases was obtained by an immunoassay detecting XMRV-neutralizing antibodies, PCR and fluorescence *in situ *hybridization; results from seven different prostate cancer patients were in concordance by all three methods [4]. Whole XMRV provirus amplification from malignant prostate tissues yielded amplicons larger than 9 kb in contrast to the full-length 8.2 kb genome. The "extra" DNA has not yet been identified, but smaller provirus amplicons were also found, indicating both internal deletions and extensions. Dr. Petros suggested that aberrant XMRV integration events and internal deletions result in substantial variation among integrated XMRV sequences in prostate cancer tissues.

In contrast, **K. Sfanos **(Johns Hopkins University, Baltimore, Maryland) and co-workers failed to detect XMRV in prostate cancer and benign tissue, pointing out no virus has been causally linked to prostate cancer despite 30 years of searching. A real-time duplex PCR assay developed in collaboration with A. Rein, NCI, Frederick, Maryland, was described. Both XMRV and CCR5 (a single copy nuclear gene) were amplified in the same PCR well, the latter confirming the quality of the DNA. As a positive control, CWR22Rv1 (an XMRV-infected prostate cancer cell line) genomic DNA was diluted into HeLa or 293T cell genomic DNA. The assay could detect ~20 copies of XMRV DNA in a vast excess of uninfected cell DNA. DNA from 161 prostate tumors was assayed and, while CCR5 DNA was detected, no XMRV-specific amplicon was obtained. IHC performed with polyclonal antisera against MoMLV p30 Capsid (CA) and gp70 Envelope surface subunit (SU) protein likewise failed to demonstrate staining of prostate tissues (596 prostate tumors and 452 benign prostate) with either antiserum. Possible reasons for the negative results were that *RNASEL *R462Q homozygotes were not selected (a finding that is inconsistent between all of the studies), that XMRV was not detected because of sequence variations, or that infected cells are present at an extremely low level and below the limits of sensitivity. Differences in PCR and serological methods between the different studies could also contribute to the different findings [7].

**N. Fischer **(University Medical Center, Hamburg, Germany) presented on the prevalence of XMRV in prostate cancer and viral mechanisms in tumorigenesis. Using RT-PCR of cryo-preserved and fresh prostate tissues, XMRV was found only rarely in sporadic prostate cancer (1/300) and in 1/70 benign controls [30]. Additionally, in collaboration with researchers at the Robert Koch Institute, Berlin, Germany, only 1/50 benign prostatic hyperplasia cases was positive using polyclonal antisera, while none of ten high grade prostate cancer cases was positive. To investigate a possible indirect mechanism of carcinogenesis involving stromal cell infections, studies with a cytokine antibody array indicated that several proteins were down-regulated in prostate stromal fibroblasts (PrSc), including TIMP1&2, IGFBP2&4, HGF, and IL-13. In contrast Gro-α was up-regulated. Interestingly, XMRV replication enhanced the migration of LNCaP cells through Matrigel. Dr. Fischer suggested that XMRV could indirectly contribute to prostate cancer through infection of stromal cells that release cytokines affecting cell invasion and tumor progression.

**B. Danielson **(Baylor College of Medicine, Houston, Texas) sought to further define the geographic distribution of XMRV among prostate cancer patients in the US by investigating the association with *RNASEL *R462Q, and searching for correlations with clinical/pathological parameters [5]. The study involved 144 prostate cancer patients from Texas, with no preoperative treatment, who underwent radical prostatectomy. Of these, 32 (22.2%) were determined to be positive for XMRV. Nested PCR was used to amplify a 650 bp region of the *env *gene, and specimens were considered positive if one or more of three PCR replicates yielded a correctly-sized amplicon. PCR products from 17 XMRV positive samples were sequenced and found to be 98.6-100% identical to XMRV VP62. XMRV DNA was detected in both normal and tumor tissues, and a correlation with the *RNASEL *QQ genotype could not be established. In addition, there was no correlation between the presence of XMRV and tumor grade. Among factors important for the detection of XMRV were the level of input DNA (650 ng) and amplification of the *env *gene (compared with *gag *and *pol*).

Additional talks summarizing prostate cancer studies included a presentation of **F. Ruscetti **(NCI, Frederick, Maryland). Antibody to XMRV Envelope protein was detected in plasma from prostate cancer patients and expressed prostate secretions (EPS). Transmission of XMRV from prostate cancer plasma and EPS to LNCaP cells in culture was demonstrated immunologically by western blotting. Transmission of XMRV from plasma from NIH prostate cancer patients to LNCaP cells was also shown by virus culture. Infectious virus and antibodies against XMRV were observed in the blood of some prostate cancer patients. Finally, virus was detected in prostate cancer plasma using a novel indicator cell line developed at the NCI (see description of K. Lee's presentation below).

**W. Switzer **(CDC, Atlanta, Georgia) reported on 162 prostate cancer patients collected at Fox Chase Cancer Center in Philadelphia, Pennsylvania. Using nested PCR on prostate tumor tissue DNA, PCR products were obtained for *gag*, *pol *and *env *from one patient, from *pol *and *env *for a second, and *pol *alone from a third (all samples were negative for mouse mitochondrial DNA). However, PCR was not successful in all replicates on individual samples (the range of XMRV-positive to total numbers of replicates was between 1/4 to 7/9). There was 4.8 to 6.5% divergence in a 167 bp *pol *sequence between the newly detected viruses and XMRV strains in the public databases, and less than 2% divergence in a 323 bp *gag *sequence. All 162 plasma samples were antibody negative using western blot testing. He also presented negative data on CFS and matched health controls that were previously published [15].

**J. Blomberg **(Uppsala University, Uppsala, Sweden) assayed DNA by qPCR from trans-urethral resections from prostate tissue of 400 patients with benign or malignant prostatic hyperplasia from Umeå University Hospital, all of which were negative. There were three posters on prostate cancer. **N. Makarova **(Emory University, Atlanta, Georgia) described an XMRV neutralizing antibody (NAb) assay. Sera from 16 of 258 prostate cancer patients screened (6.2%) were positive for XMRV Nab, which is lower than in their original study [4]. **Y. Ikeda **(Mayo Clinic, Rochester, Minnesota) showed a number of XMRV-positive biopsy samples using nested PCR for *gag *(1 of 40 normal/benign, 4 of 70 intermediate prostate cancers, and 1 of 40 high-grade prostate cancers at the Mayo Clinic). However, no XMRV-specific 24 bp deletion was found in the *gag *leader regions of the PCR-positive clinical samples. **J. Das Gupta **(Cleveland Clinic, Cleveland, Ohio) described a novel qPCR assay for detecting XMRV RNA in urine. About 26% of prostate cancer cases (31/120) were XMRV positive, while 1/22 urine specimens (4.3%) from normal healthy control individuals was XMRV positive. Urine samples were negative for mouse mitochondrial DNA.

### XMRV and Chronic Fatigue Syndrome

Pointing out that mouse cells contain ~50 copies each of endogenous MLV DNA, and that less than one cell's worth could yield a detectable PCR product, **B. Huber (**Tufts University, Boston, Massachusetts) emphasized the urgent need to distinguish contaminating mouse sequences from true XMRV infections. PBMC DNA was tested for XMRV by qPCR and nested PCR, using primers specific for regions of the XMRV *pol *and *gag *genes, respectively. In addition Dr. Huber's group assessed potential mouse DNA contamination using both qPCR for murine mitochondrial cytochrome oxidase and/or conventional PCR for IAP DNA. While control experiments verified the sensitivity of all assays, her group failed to detect XMRV in 184 CFS patients and 25 healthy controls. However, positive results were obtained with some samples using the *gag *nested PCR assays. DNA sequencing of the PCR products revealed sequences identical to those described from prostate cancer and CFS patients, in addition to sequences more closely related to endogenous MLVs. However all samples testing positive for XMRV or MLV DNA were also positive for mouse IAP and mitochondrial DNA, using either assay. The source of this apparent contamination is under investigation.

Contrasting data was subsequently presented by **M. Hansen **(Cornell University, Ithaca, New York), who summarized a blinded study ("10/10/10" study) designed to determine whether XMRV could be detected in PBMCs from three small groups of subjects from a single geographic area. Study subjects (10 per group) were classified as severely ill with, or recovered from, CFS. A control group lacked a CFS diagnosis at any time. XMRV RNA was evaluated by nested RT-PCR, using *gag *primers [1]. In addition, PCR with mouse mitochondrial DNA primers were used on all cDNA preparations to exclude mouse cell contamination. *Gag *sequences similar to polytropic MLV were detected in 8 of the severely-ill CFS patients, 3 of those who had recovered, and one of the controls. In order to determine whether infectious virus could be recovered, a subset of these blood samples was incubated with LNCaP cells followed by serial passage over several weeks. PCR analysis revealed that cultures exhibiting *gag *sequences corresponded to those inoculated with CFS patient plasma. Although a relatively small study, the prevalence of virus in severe or recovered CFS patients (55%) relative to the control group (10%) strengthened the findings of Lombardi *et al*. [3].

Supporting the results of the Cornell study, **S.C. Lo**, (FDA/CBER, Bethesda, Maryland) reviewed his previously published findings on the presence of MLV-related virus gene sequences in both CFS patients and healthy controls [31]. A unique feature of this study was that portions of the CFS blood samples had been maintained in frozen storage at -80°C from the mid 1990s. Using nested PCR, MLV-like *gag *gene sequences could be amplified from PBMC DNA in 32 of 37 patients meeting the accepted diagnostic criteria for CFS (86.5%) compared with only 3 of 44 (6.8%) healthy volunteer blood donors. This study also detected viral RNA in the frozen plasma samples of these CFS patients. However, *gag *and *env *sequences from CFS patients were more closely related to those of polytropic mouse endogenous retroviruses than to XMRV. Recognizing the increasing concerns of contamination (including the PCR primers themselves, laboratory reagents or commonly used viral vectors), semi-nested PCR was used to demonstrate the absence of mouse mitochondrial DNA. Dr. Lo pointed out in his concluding statements that additional studies are needed to determine whether MLV-related viruses have a causal or secondary role in the development of either CFS and prostate cancer.

Two European studies failed to detect XMRV infection in CFS and MS patients. A study presented by **N. Bannert **(Robert Koch-Institute, Berlin, Germany) failed to detect the presence of antibodies against *gag *and *env *in serum from 36 CFS patients (Fukada/CDC criteria), 50 multiple sclerosis patients (fatigue severity scale 4,7+/-1.07) and 17 healthy individuals. In addition XMRV was not detected in DNA isolated from stimulated PBMCs of 39 CFS, 50 MS and 30 healthy controls using a nested PCR, and reverse transcriptase activity was absent from the supernatant from stimulated PBMCs. Co-cultivation of PBMCs from a subset of patients with LNCaP indicator failed to recover infectious virus.

**J. Blomberg **(Uppsala University, Uppsala, Sweden) investigated 50 CFS patients (Fukada criteria) using virus isolation with LNCaP cells with patient plasma as inoculum from 40 of these patients. Cultures were monitored at day 5 with *integrase *qPCR, potential positive cultures were passed for another 5 days. Though three cultures were initially positive with a few copies, only one could be passed twice, but not further. The other two initially positive cultures lost signal after the first passage. Virus was not recovered. Serological testing was performed on 60 CFS samples and 100 blood donors using a multi-epitope approach with 22 peptides spanning Gag and Envelope coated on Luminex beads. Peptides were designed to react broadly by conserved sequence selection and inclusion of degenerate amino acids. Sera with a reaction above background (non-coated beads) against minimally three peptides were considered positive. One blood donor sample and two CFS samples reacted in this fashion. The authors concluded that XMRV and related viruses are rare in Sweden.

A poster of **Blanco ***et al*. (Irsi Caixa Foundation, Barcelona, Spain) used an alternative approach to look for XMRV. PBMCs from patients were immortalised by infection with Epstein Barr virus, DNA extracted from cell pellets and tested for XMRV using real time PCR for *pol *(50-nt) and two nested PCR assays for *gag *and *env *genes. Eleven CFS patients (Fukada and Canadian criteria) and 5 healthy donors were tested. Three CFS patients and one control were found positive in the nested *env *approach, one CFS patient and one control in the *gag *nested PCR, and four CFS patients and two controls in the real time *pol *PCR assays. Sequencing of the three *gag *amplicons found the 24-nt deletion characteristic of XMRV. The authors concluded that EBV-transformed cell lines can harbour XMRV specific sequences.

The final presentation of the CFS session was delivered by **J. Mikovits **(Whittemore Peterson Institute, Reno, NV) who shared data on a recent study detecting XMRV in the peripheral blood of CFS patients in the United Kingdom. All study patients (50) met the requirements for CFS based on rigorous criteria. Peripheral blood from these patients was shipped to NCI-Frederick for plasma and PBMC isolation, after which serology and virus isolation were performed at two different laboratories. A multi-faceted approach involved (i), nested RT-PCR for *gag *or *env *sequences (ii) detection of Env antibodies in plasma (iii), Western analysis from LNCaP cells co-cultured with subject's cell-free plasma and (iv), immunological detection of viral proteins expressed by activated PBMCs. Collectively, this study indicated the presence of infectious virus in >60% of CFS patients. XMRV could be transmitted either cell-associated or cell-free from both activated lymphocytes and plasma from infected individuals by passage to LNCaP. Maintaining that the worldwide distribution of XMRV is greater than previously assumed, Dr. Mikovits concluded her presentation by calling for additional studies addressing the replication and pathogenesis of XMRV in the human population, as well as prioritizing the development of antiviral agents for testing in the appropriate clinical setting.

### Development of XMRV Diagnostic Tools

A central theme of the Workshop was the availability to the research community of reliable diagnostic reagents for nucleic acid testing, serology and virus culture. Additionally, there was general consensus among attendees for including sensitive PCR methods to detect contaminating MLV-related and mouse DNA. Based on the ability to recapitulate a non-human primate model of XMRV infection [21], **X. Qiu (**Abbott Diagnostics, Illinois) presented an update on their collaboration with researchers at Emory University and the Cleveland Clinic to develop a series of high-throughput immunoassays for future epidemiological studies. Using serum from XMRV-inoculated rhesus macaques and a viral lysate as source of antigen, antibody responses were detected for surface subunit (SU) Envelope protein, 9 days post-inoculation, followed by the trans-membrane protein p15E (TM) at Day 11 and Capsid (CA) at Day 14. Chemiluminescence-based indirect (anti-human) and direct (double antigen) assay formats based on each of these antigens are currently under development. By changing the source of SU from a bacterial to a mammalian expression system and incorporating Avidin Biotin Complex signal amplification, sensitivity of the SU immunoassay was improved >1000-fold. The prototype, direct SU assay provided substantial discrimination between a blood donor negative population and the 29 XMRV seropositive primate bleeds.

**R. Bagni **(SAIC-Frederick, Frederick, Maryland) summarized current NCI efforts to develop serological reagents for XMRV. In the absence of a *bona fide*, pedigreed antibody-positive clinical control, a "training set" of 116 samples, 39 of which were designated XMRV-positive from the Lombardi *et al*. study, were examined for the presence of XMRV-specific antibodies. Of the 9 candidate XMRV proteins, a strong serological response to the SU and TM, and CA was observed, while to a lesser extent, antibodies to p12, MA and NC could be detected. Dr. Bagni also introduced the concept of a "positivity algorithm", i.e. the number of XMRV antigens for which an immunological response must be detected before designating a sample positive. A total of 64 expression clones constructed for development of the NCI XMRV ELISA has now been deposited at the NIH AIDS Research and Reference Reagent Program https://www.aidsreagent.org to be accessed by researchers of the extramural community.

As a valuable complement of nucleic acid and serological assay reagents, **K. Lee **(NCI, Frederick, Maryland) described the development of a novel cell line to rapidly assess XMRV or XMLV replication. **D**etectors of **E**xogenous **R**etroviral **S**equence **E**lements, or DERSE indicator cells, exploit a specialized retroviral vector containing an inverted, intron-interrupted green fluorescent protein (GFP) reporter cassette. Although GFP is not expressed within a target cell after an initial infection, transfer of an intron-less vector to new cells during a second round of XMRV infection permitted GFP expression, which can be easily monitored by microscopy. Importantly, the DERSE cell line permits virus detection in a little as three days, representing a considerable time saving over standard methods. Dr. Lee indicated that this cell line will be deposited in the NIH AIDS Reagent Repository for use by researchers in the extramural community. While clearly not intended for first-line (high throughput) analysis, the DERSE cell line will most certainly find use as a confirmatory strategy.

**M. Kearney **(NCI, Frederick, Maryland) presented two highly-sensitive single-copy assays for detection of both XMRV and MLV-related viruses in blood products. The first of these, the XMRV single copy assay, or X-SCA, is a qualitative PCR assay (analogous to that employed for HIV detection) capable of detecting a single pelletable virion in plasma or XMRV DNA in whole blood or PBMC. As a complement, the XMRV single genome sequencing assay (X-SGS) likewise facilitates individual sequencing of large genomic fragments. Preliminary data indicated that X-SCA compared favorably in specificity and sensitivity with related protocols under development at the FDA, CDC, Whittemore-Peterson Institute and Blood Systems Research Institute. In combination, X-SCA and X-SGS are capable of discriminating between XMRV and contaminating mouse endogenous viruses with 100% accuracy.

The concluding presentation of **G. Simmons **(Blood Systems Research Institute, San Francisco, California) set the stage for discussing future actions to help resolve disparate results presented during the Workshop. The Blood XMRV Scientific Research Working Group (Blood XMRV SRWG) was established as a National Heart, Lung and Blood Institute (NHBLI) coordinated working group to design and coordinate collaborative studies to standardize existing assays and investigate the prevalence of XMRV in blood donors. A four-phase, multi-center study was described, wherein Phase 1 involved PCR testing, in a blinded fashion, of analytical performance panels comprising pedigreed negative blood and plasma spiked with serial dilutions of XMRV infected cells and supernatants, respectively. In general, there was good agreement between participating laboratories. Phase II will compare XMRV nucleic acid detection in frozen PBMCs, whole blood and plasma from CFS patients previously identified as viremic. Importantly, replicate blood specimens would be processed at different storage intervals to determine whether the 2-4 day processing period common to many blood donor repositories affects assay performance. Phases III and IV will extend these studies to begin to examine the prevalence of XMRV in blood donors by both nucleic acid and serological methods.

### The Path Forward - Consensus and Caution

Considering the discrepancies between the different studies regarding the prevalence of XMRV, it became abundantly clear that reaching consensus on protocols for PCR amplification, for discriminating between XMRV and contaminating mouse endogenous viruses, and sample storage and processing should be an immediate priority among groups studying XMRV. The studies of the Blood XMRV SRWG are likely to be of great importance in developing such a consensus. The availability of analytical performance panels comprising pedigreed samples would also be an enormous benefit to researchers. The scientific community might also consider establishing a "repository" where protocols can be publicly deposited and compared, which could reveal nuances underlying the discrepancies observed when similar reagents are used by different groups.

Finally, there was vigorous discussion about the use and timing of interventions targeted against XMRV in CFS and prostate cancer patients. Although a small number of workshop participants advocated the immediate use of antiretrovirals that have successfully controlled HIV infection, and while a well-controlled, randomized clinical trial should not be ruled out, proceeding with caution was emphasized. Until (a) a causal role for XMRV in CFS or prostate cancer is firmly established (b), objective biomarkers are defined, and (c) uniformly-accepted assays to monitor effects on virus replication are in place, the off-label use of antiretrovirals and anecdotal reports of their efficacy will be unacceptable to third party payers/regulators, and could potentially keep valuable therapies out of reach of many patients.

## Competing interests

SL, CB and JS have no competing interests. RHS: patents, Abbott Diagnostics. RHS consulting: Abbott Diagnostics

## Authors' contributions

All authors contributed to the writing and editing of this manuscript. The final version of the manuscript was approved by all authors.
